# A novel affordable user interface for robotic surgery training: design, development and usability study

**DOI:** 10.3389/fdgth.2024.1428534

**Published:** 2024-07-30

**Authors:** Alberto Neri, Mara Coduri, Veronica Penza, Andrea Santangelo, Alessandra Oliveri, Enrico Turco, Mattia Pizzirani, Elisa Trinceri, Domenico Soriero, Federico Boero, Serena Ricci, Leonardo S. Mattos

**Affiliations:** ^1^ Biomedical Robotics Lab, Advanced Robotics, Istituto Italiano di Tecnologia, Genoa, Italy; ^2^ Department of Computer Science, Bioengineering, Robotics and Systems Engineering (DIBRIS), University of Genoa, Genova, Italy; ^3^ Simulation and Advanced Education Center, University of Genova, Genova, Italy; ^4^ Humanoid and Human Centred Mechatronics (HHCM), Istituto Italiano di Tecnologia, Genoa, Italy; ^5^ Department of Information Engineering and Mathematics, University of Siena, Siena, Italy; ^6^ Fos SpA, Genova, Italy; ^7^ Unit of Surgical Oncology, IRCCS Policlinico San Martino, Genoa, Italy

**Keywords:** surgical simulator, surgical robot, virtual reality, teleoperation, medical training

## Abstract

**Introduction:**

The use of robotic systems in the surgical domain has become groundbreaking for patients and surgeons in the last decades. While the annual number of robotic surgical procedures continues to increase rapidly, it is essential to provide the surgeon with innovative training courses along with the standard specialization path. To this end, simulators play a fundamental role. Currently, the high cost of the leading VR simulators limits their accessibility to educational institutions. The challenge lies in balancing high-fidelity simulation with cost-effectiveness; however, few cost-effective options exist for robotic surgery training.

**Methods:**

This paper proposes the design, development and user-centered usability study of an affordable user interface to control a surgical robot simulator. It consists of a cart equipped with two haptic interfaces, a VR visor and two pedals. The simulations were created using Unity, which offers versatility for expanding the simulator to more complex scenes. An intuitive teleoperation control of the simulated robotic instruments is achieved through a high-level control strategy.

**Results and Discussion:**

Its affordability and resemblance to real surgeon consoles make it ideal for implementing robotic surgery training programs in medical schools, enhancing accessibility to a broader audience. This is demonstrated by the results of an usability study involving expert surgeons who use surgical robots regularly, expert surgeons without robotic surgery experience, and a control group. The results of the study, which was based on a traditional Peg-board exercise and Camera Control task, demonstrate the simulator’s high usability and intuitive control across diverse user groups, including those with limited experience. This offers evidence that this affordable system is a promising solution for expanding robotic surgery training.

## 1 Introduction

The annual number of robotic surgical procedures performed worldwide continues to increase rapidly (1). The benefits of a minimally invasive access to the surgical field are combined with increased manoeuvrability, precise movements, an immersive 3D view and ergonomic teleoperated procedures executed from a remote console (2). However, the core of the surgery still relies on the surgeon’s degree of expertise and experience, making the outcome of the surgery vary according to the surgeon’s skills. Robotic surgical skills are unique and not derivative from open or laparoscopic surgery (3). As demonstrated in laparoscopy, relocating the place for acquiring essential skills from the operating room to the simulation laboratory offers notable benefits for trainees, hospitals, and patients (4).

Challenges associated with the use of real surgical robotic systems for training practice include cost, low availability due to extensive clinical utilization, and the risk of equipment damage. A solution relies on computer-based or virtual reality (VR) simulators designed specifically for robotic surgery. VR training for robotic skills acquisition is described in the literature as early as 2007 (5, 6). In recent years, simulation has expanded into the surgical area as a safe and cost-effective method for training (7). In fact, it provides a VR environment for novice robotic surgeons to practice surgical skills without compromising patient safety (8). While the simulated procedure cannot fully replicate the intraoperative experience, it serves as a crucial component in the training of surgical professionals. The primary goal remains skill acquisition, which, when optimized, could enhance outcomes and patient safety (9).

As stated in (7), there are five VR simulators available for robot-assisted surgery in the field of urology: the Da Vinci Skills Simulator (89,000 USD), the Mimic dV Trainer (158,000 USD), the Simsurgery Educational Platform simulator (62,000 USD), the Robotic Surgical Simulator (120,000 USD) and the RobotiX Mentor (137,000 USD). Additionally, a more affordable solution has recently been introduced, namely BBZ’s LEO simulator (40,000 USD) (10). However, most of these simulators cannot fully meet demand due to price barriers (11). While the average cost of a simulator may be affordable for hospitals, the same may not hold true for all universities or medical schools aiming to offer training in robotic surgery. Indeed, as remarked in (7), the cost-effectiveness of simulators merits consideration. A systematic review of the effectiveness of simulation in urology indicates that certain low-fidelity simulators are considered more cost-effective than their high-fidelity counterparts (12).

As noted by (13), the concept of utilizing low-cost simulations has been in practice for several years. One of the first exercises developed is knot tying (14). They used low-cost and easily accessible materials to develop a cost-effective curriculum, demonstrating construct validity. Recently, (15) introduced a cost-effective chest tube simulator. They used a 3D printer to replicate the human chest cavity and facilitate the practice of closed chest drainage techniques. (16) have developed a simulator for neurosurgery, specifically for cerebrovascular bypass surgery. The authors constructed a low-cost, reusable, high-fidelity simulator utilizing an anatomical skull and brain model, artificial vessels, and a water pump to mimic extracranial and intracranial circulations. An interesting example in the field of open surgery and with a cost of less than 1 USD is (17). They developed a training simulator for open dismembered pyeloplasty using a catheter tip syringe filled with 30 mL of air, tape, a 260 modeling balloon (the urether), and an 11-inch party balloon (the dilated renal pelvis). Moving to laparoscopy, while numerous low-cost laparoscopic box trainers are available for traditional, non-robotic laparoscopy (18, 19), the same does not hold true for robotic laparoscopy.

Few attempts were made to develop cost-effective robotic surgery simulators, possibly due to the availability of commercial solutions. The architecture proposed by (6) utilizes a PC screen and two Phantom Omni devices (an earlier version of Geomagic Touch) equipped with an electromechanical gripper to simulate the surgeon’s master console. The control of the simulated robotic arms relies on inverse kinematics equations. They created a bean drop task and visualized the scene using the OpenSceneGraph rendering library. (20, 21) employed a similar architecture, utilizing a VR visor and two Phantom Omni equipped with the standard stylus. Using V-REP (Coppelia), they designed various surgical training tasks, including pick and place, peg board, and suturing. Lastly, (22) proposes a different approach using two Razer Hydra game controllers to control the simulated robotic arms within a Unity environment. The exercise developed is a bead move task. None of these solutions incorporate pedals, commonly utilized in surgical robots for controlling robotic arm functions. Moreover, none aim to accurately replicate the console’s ergonomics to ensure that the surgeon’s head, shoulders, and arms maintain the same positioning as in actual surgery. Finally, no one proposes a usability study with surgeons and a control group to evaluate the effectiveness of the offered solution.

**Figure 1:**
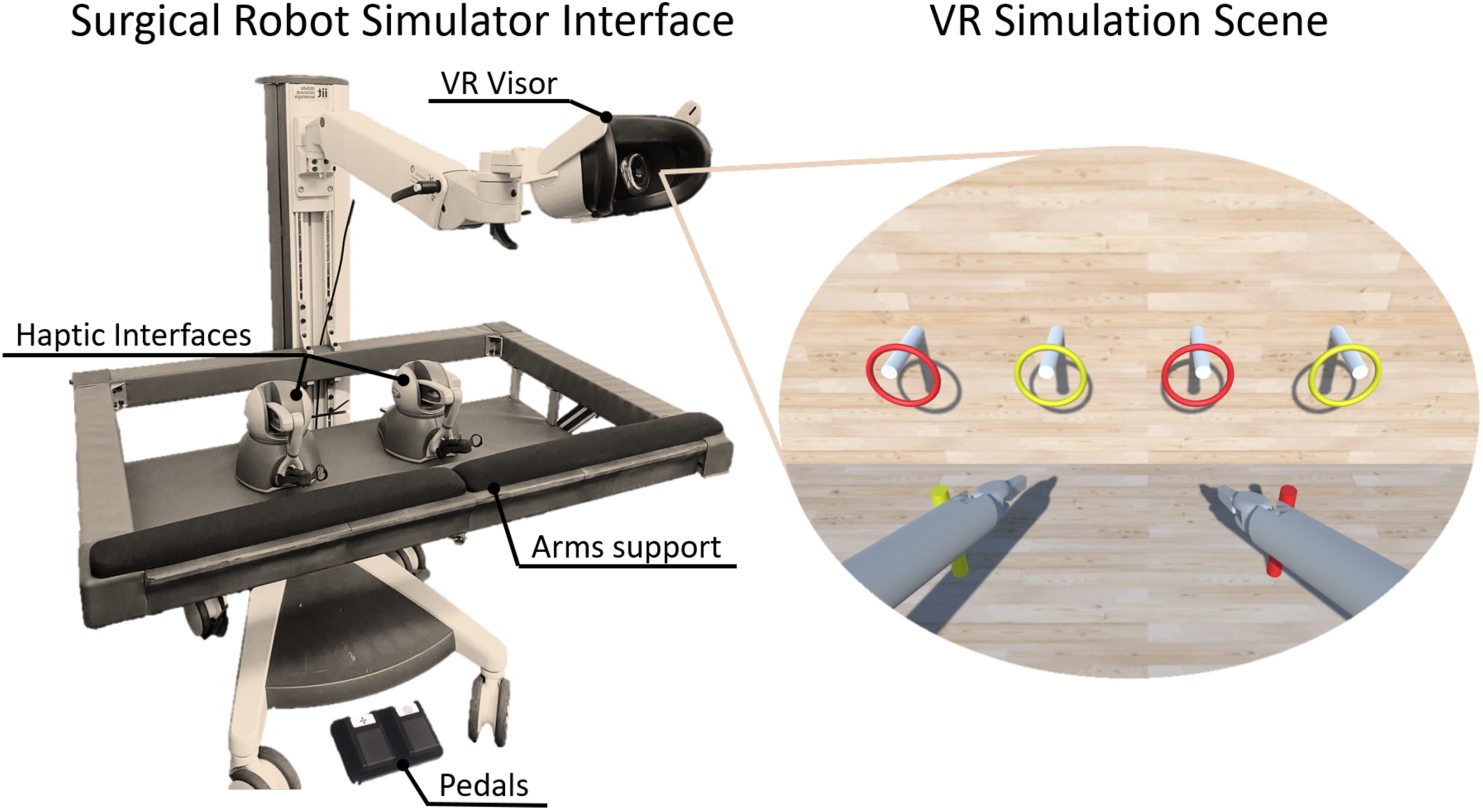
The proposed surgical robot simulator. The interface consists of two haptic devices, two USB pedals and a VR headset displaying the simulation scene.

To face these needs, inspired by (21), we propose the design, development and user-centered usability evaluation of an affordable user interface to control a surgical robot simulator, shown in Figure 1. The system was designed to be simple, scalable and cost-effective. A cart was structured to integrate all the devices necessary to control the robotic surgical simulator: two haptic interfaces, two pedals and a VR Visor. A software interface was developed to enable the teleoperation of the simulated robotic instruments. In addition, we introduced the possibility of enabling haptic feedback in case of collisions between the simulated grippers and the scene elements. Finally, we developed two basic exercise tasks exploiting Unity. Its flexible development environment enables the creation of intricate scenes, incorporating diverse components such as robots, tools, physics, and organs to enhance the simulator further.

The major contribution of this work lies in its validation through a comprehensive usability study involving three distinct groups of subjects: controls, robotic surgeons and non-robotic surgeons. The study’s primary goal was to evaluate performance differences between controls and robotic surgeons. For this, we recruited 13 trained surgeons experienced in robotic surgery, while for the control group, we selected 23 inexperienced subjects without any previous experience in surgery and robotics. We then aimed to determine if we could discern performances between robotic and non-robotic surgeons. To explore this hypothesis, we recruited a new subset of 6 surgeons with no prior experience in robotic surgery. Lastly, despite the absence of haptic feedback in current robotic surgery, debates on its utility remain active and unresolved in the literature (23, 24). Therefore, given the option to include haptic feedback in our simulator, we integrated it into an exercise to conduct a pilot study. The objective was to assess whether performance differs when the task is executed with or without it.

In summary, we have maintained the advantages of previously proposed surgical robot simulators while incorporating the missing parts, such as the physical interface and pedals. Then, we evaluated it with a comprehensive usability study involving both surgeons and controls.

The article is structured as follows. The main technical features of the system are detailed in Section 2. The experimental validation and usability study are presented in Section 3. The results and discussions are outlined in Section 4. Finally, the conclusions are reported in Section 5.

## 2 Material and methods

### 2.1 Surgical robot simulator console cart

**Figure 2:**
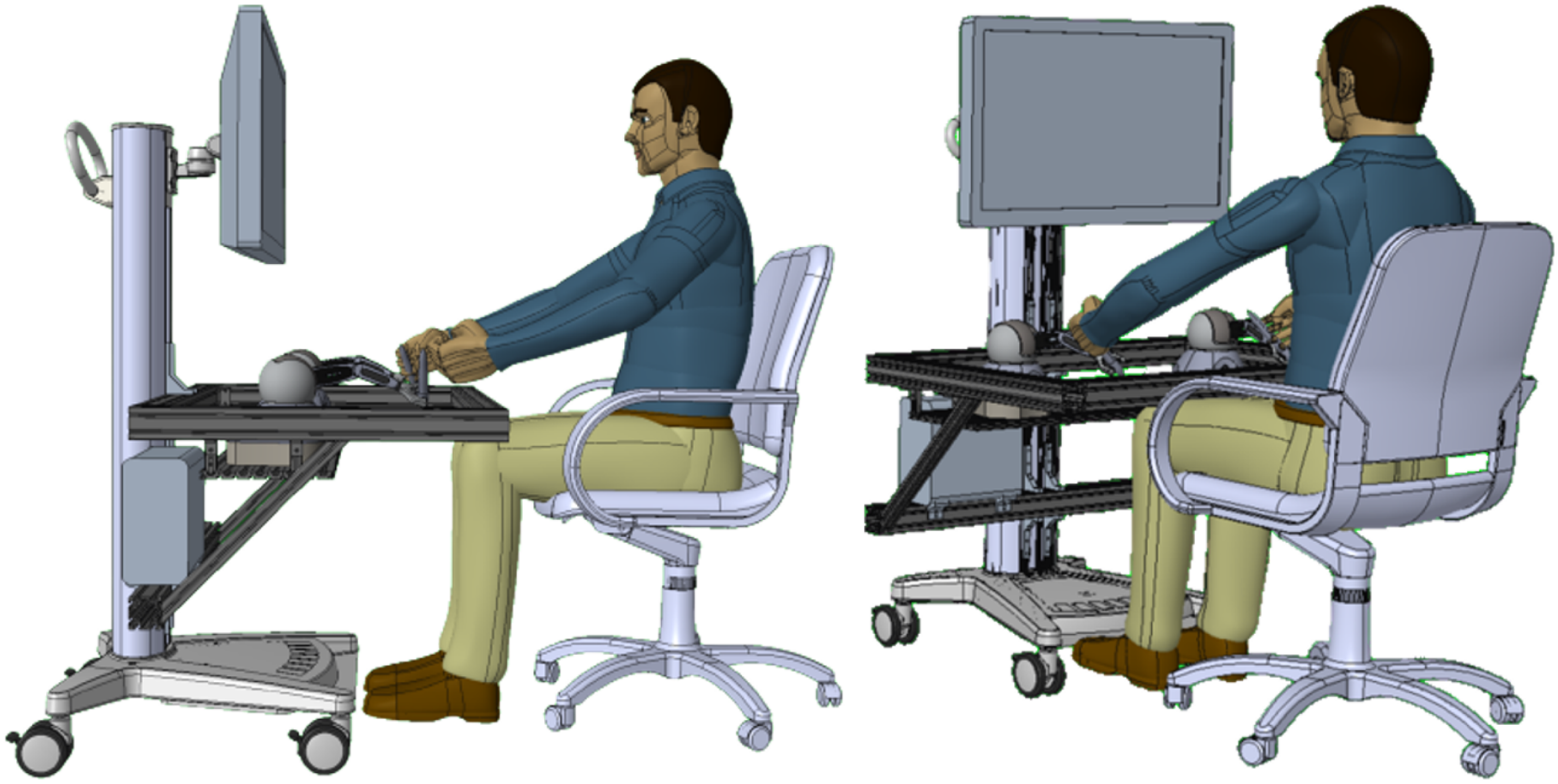
CAD design of the surgical robot simulator interface, which can be equipped with a 2D/3D monitor or with a VR visor.

A cost-effective console was developed to integrate all the required devices for control of the surgical robot simulator, as shown in Figure 2. The design was based on the regulations for Video-Display-Terminal workstations (ISO 9241) to guarantee an ergonomic seat, such as (i) desk or support for the surgeons’ arms at the height of 74cm±2cm, (ii) support arms depth from 10 cm to 20 cm, (iii) adjustable chair to allow the regulation of the seat height, and (iv) distance between the eyes and the 2D/3D monitor around 50–70 cm or an adaptable arm holding the VR visor. Although the two setups are different in terms of ergonomics, they are both exploited by surgical robots on the market. For example, Da Vinci exploits an immersive visual interface similar to VR, while Hugo (Medtronic) and Versius (CMR Surgical) use a display setup. The haptic devices’ encumbrance and workspace have been taken into account in order to be able to handle the grippers at a distance of 15 cm from the arm support while keeping the wrist free for movement. To meet these specifications, a standard commercial cart was customized by incorporating arm supports and a shelf using aluminium bars. The console was equipped with:

• Two haptic interfaces used as master manipulators to teleoperate the simulated robotic instruments and the endoscope. Each haptic device was outfitted with a gripper interface (Twee, BBZ s.r.l, Italy) to provide an additional degree of freedom to control the forceps of the robotic surgical instruments;
• A VR visor or a 3D monitor for stereoscopic visualization of the simulated scene;
• Two USB pedals for control of the instruments and the camera motion.

### 2.2 Surgical robot simulator software architecture

**Figure 3:**
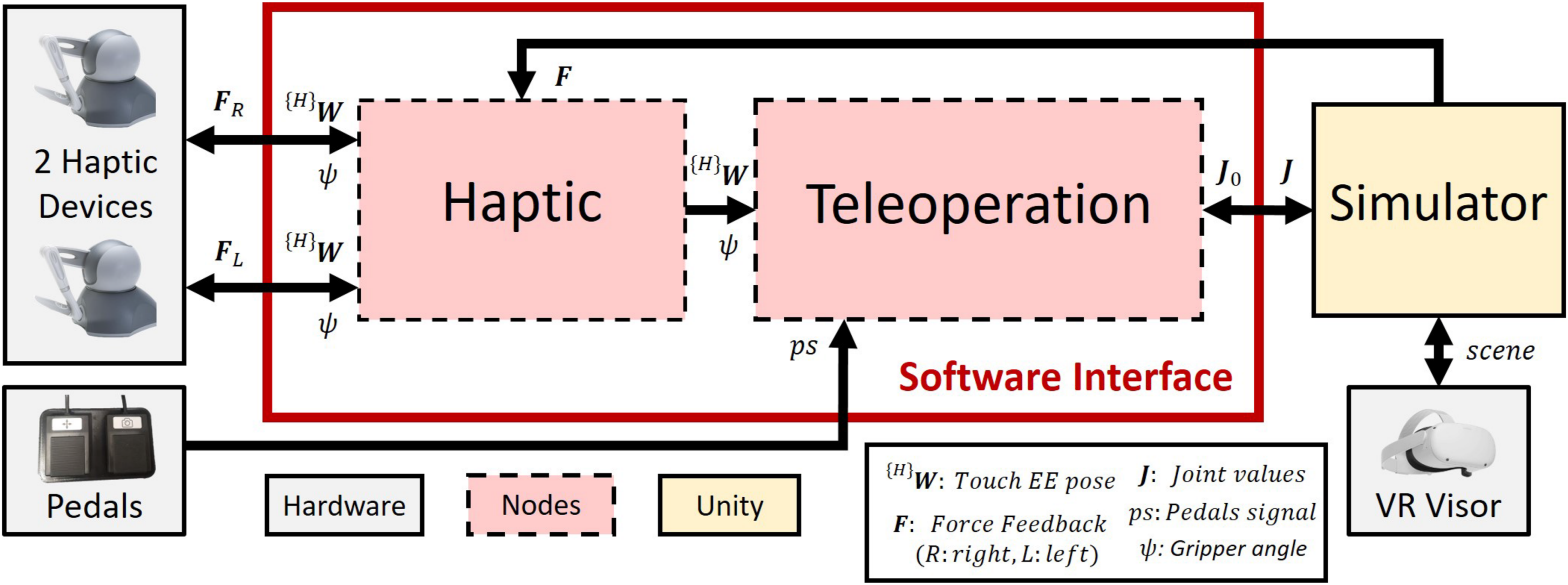
Surgical robot simulator architecture. The Haptic node retrieves the haptic device data (^(*H*)^**W** and ψ) and sends them to the Teleoperation node, which controls the robotic arms’ movements in the simulation (Section 2.2.3). The Unity scene is shown through the VR, and the haptic feedback forces (Section 2.3.1) are directly sent to the Haptic node.

Figure 3 shows the Surgical Robot Simulator architecture. A software interface node Haptic is implemented to retrieve position and orientation data from two haptic devices. Using this input data along with the signal from the pedals, the Teleoperation node manages the high-level control of a robotic surgical system, simulated in Unity (25), and displayed through the visor. Finally, the Haptic node gets the interaction forces information from the simulation and provides haptic feedback to the manipulators. The two nodes are kept separate to ensure greater modularity. This allows for parts of the architecture to be reused for other purposes, as demonstrated in (26).

#### 2.2.1 Pedals

**Figure 4:**
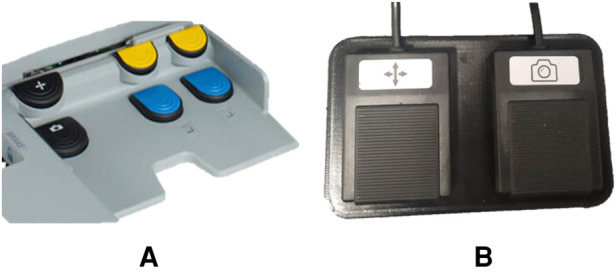
Da Vinci robot pedals set (**A**) compared the proposed pedals set (**B**).

Foot pedals are required to switch between the control of the robotic arm and the camera control. The pedal set of a Da Vinci robot consists of 7 pedals (Figure 4A). The yellow and blue pedals on the right are used to carry out the coagulation (blue) and cutting (yellow) operations. Considering that this project does not currently implement the simulation of these functionalities, only the two black pedals (Figure 4B) have been replicated, in particular:

• The pedal with the camera symbol is used to activate the robotic camera control. The simulator allows controlling the camera through the haptic device’s movement by holdingdown this pedal.
• The pedal with the compass symbol is a clutch used to deactivate the two instruments, allowing the surgeon to reposition the manipulators in the preferred position. In order to activate this feature, the user needs to holddown the pedal.

As shown in Figure 3, when the user presses either pedal, a signal (ps) is sent to the Teleoperation node.

#### 2.2.2 Haptic device software interface

**Figure 5:**
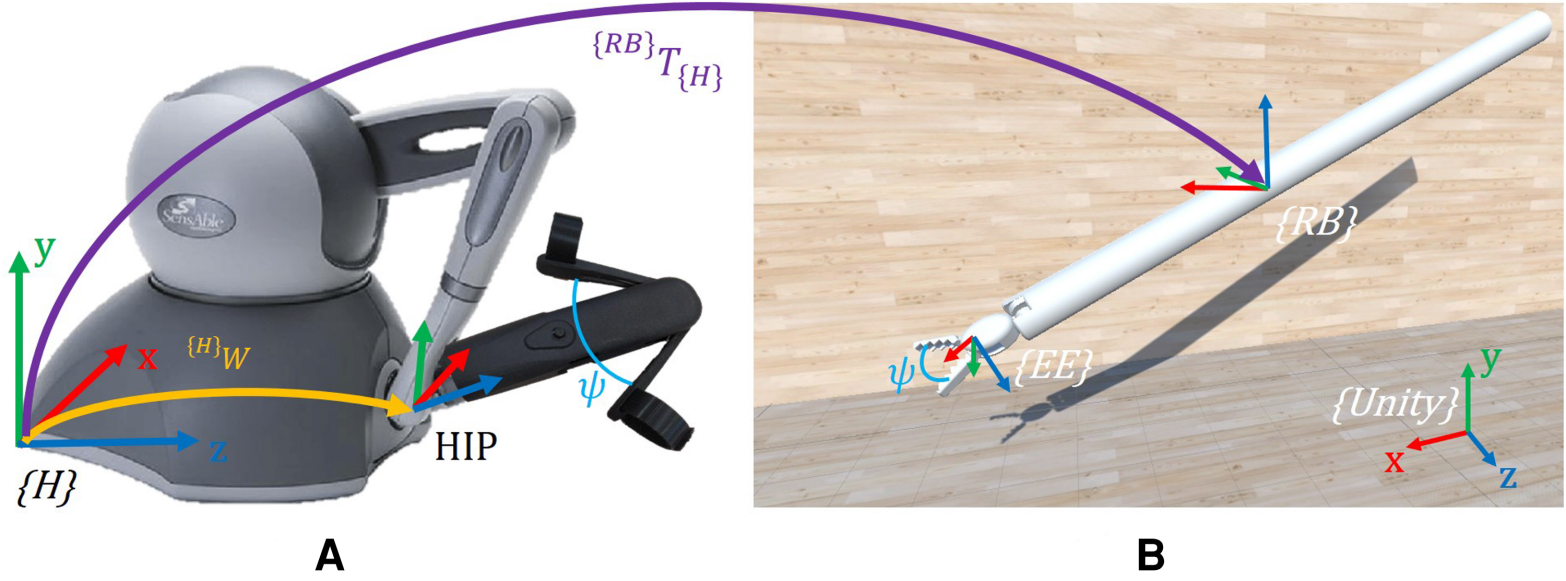
Description of the reference systems adopted. (**A**) Haptic interface reference system {H}. (**B**) Simulated robotic instrument. Here, only the Endo-Wrist part of the Patient Side Manipulator (PSM) is present in the simulation. {Unity} is the Unity world reference system, {RB} the robot base, and {EE} the end-effector. ^{*RB*}^**T**_{*H*}_ is the transformation matrix between {H} and {RB}. ^{*H*}^**W** is the pose of the HIP in {H}. ψ is the gripper opening angle, which is reproduced in the simulated instrument. Note that in Unity, the reference systems are left-handed.

Observing Figure 3, the Haptic node serves as the software interface that retrieves the raw position and orientation of the haptic device’s end-effector, as well as the gripper’s opening angle and sends all of them to the Teleoperation node. Moreover, the Haptic node receives force feedback reference signals from the Simulator node, which are then sent to and rendered by the haptic devices. Figure 5A shows the homogeneous matrix ^(*H*)^**W** representing the pose of the end-effector, also called Haptic Interface Point (HIP), in {H}; ψ is the gripper’s opening angle. In order to eliminate noise in the opening angle measurement, a software calibration was implemented to remove the offset of ψ, setting the minimum value to $0^{\circ }$ when the gripper is closed.

#### 2.2.3 High-level teleoperation control

The Teleoperation node is implemented to replicate the movements of the haptic interfaces on the robotic surgical instruments. In the simulation, in order to simplify and generalize the representation of the surgical robotic system, two generic instruments, similar to the Endo-Wrist of the Da Vinci Patient Side Manipulator (PSM) (27), are used (Figure 5B).

Resembling the Da Vinci, each PSM is a 7-DoF actuated arm that moves a surgical instrument around a Remote Center of Motion (RCM). The first 6 DoFs correspond to Revolute (R) or Prismatic (P) joints combined in an RRPRRR sequence. The last DoF corresponds to the opening and closing motion of the gripper. The end-effector (EE) is defined as the simulated gripper’s fulcrum (Figure 5B). The standard Da Vinci DH parameters (21) are used to define the transformation between the robot base {RB} and the {EE} (see Table 1).

**Table 1:** DH parameters of the PSM.

Link	Joint	$a_{i}$	$\alpha_{i}$	$d_{i}$	$\theta_{i}$
1	R	0	−π/2	–	$q_{\;p,1}$
2	R	0	−π/2	–	$q_{\;p,2}$
3	P	0	0	$q_{\;p,3}$	–
4	R	0	π/2	–	$q_{\;p,4}$
5	R	$a_{5}$	−π/2	–	$q_{\;p,5}$
6	R	0	−π/2	–	$q_{\;p,6}$

The robotic tool is initialized with the initial joint values ($J_{0}$), sent by the Simulator node (Figure 3), and its teleoperation is governed by the logic described in Section 2.2.1 and the associated ps signal. The pose of the EE is updated using an incremental approach, where the real-time EE pose is continuously adjusted by adding the incremental relative movements, namely the variation in position and orientation of the HIP, as follows:

(1) $${\;}^{\left\{RB\right\}}P_{1}{=}^{\left\{RB\right\}}\Delta W{*}^{\left\{RB\right\}}P_{0}$$

where ${\;}^{\left\{RB\right\}}P_{1}$ and ${\;}^{\left\{RB\right\}}P_{0}$ are the homogeneous matrices describing the final and current pose of the EE, with respect to the {RB} reference system. ${\;}^{\left\{RB\right\}}\Delta W$ describes the pose variation of the HIP in {RB} and is defined as:

(2) $${\;}^{\left\{RB\right\}}\Delta W{=}^{\left\{RB\right\}}T_{\left\{H\right\}}*{(}^{\left\{H\right\}}W_{1}{*}^{\left\{H\right\}}W_{0}^{-1})$$

where ${\;}^{\left\{RB\right\}}T_{\left\{H\right\}}$ is the rigid transformation between {H} and {RB}, while ${\;}^{\left\{H\right\}}W_{1}$ and ${\;}^{\left\{H\right\}}W_{0}$ are the final and initial pose of the HIP in {H}. All variables used are in the form of homogeneous matrices.

Exploiting a software library for inverse kinematics calculations and specifying ${\;}^{\left\{RB\right\}}P$ as the goal pose, we can determine the desired joint values (J). J will then be transmitted to the Unity simulation.

#### 2.2.4 Robotic camera control

**Figure 6:**
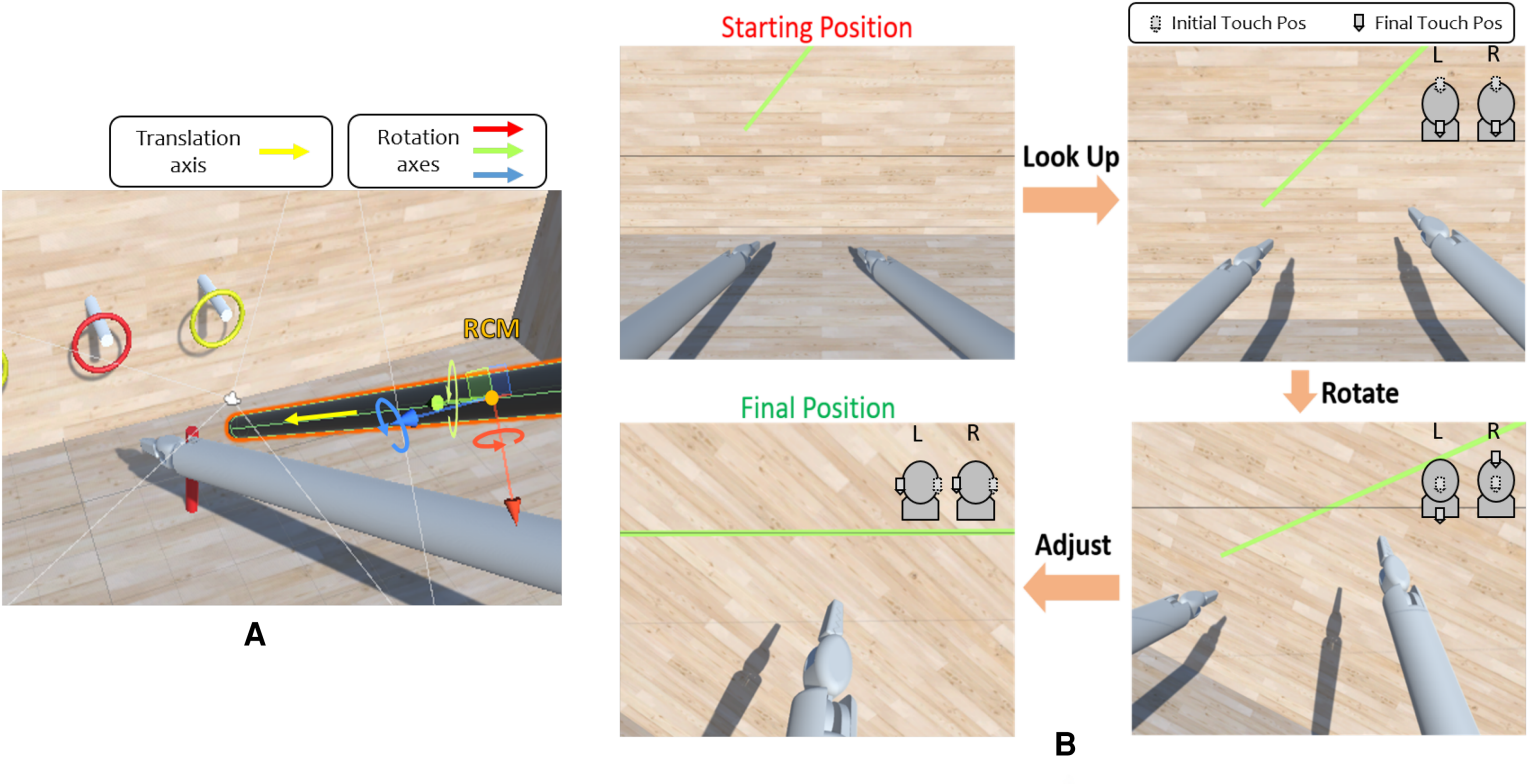
(**A**) Robotic camera degrees of freedom. The camera can rotate around the three axes of its RCM and move along its own body axis. (**B**) Example of camera control task. These are possible camera movements to perform the exercise. The task is completed when the user is satisfied with the obtained alignment. At the top right of each image, there is an example of how the left (L) and right (R) Touch end-effectors can be moved to achieve the desired camera orientation. The dotted EE indicates the starting position. For instance, in the top right image, moving both the EEs down leads the camera to look up. In the bottom right image, moving one EE up and the other down results in a rotational movement.

The Robotic Camera (RCam) is a 4-DoF actuated arm, which moves the camera about its RCM through revolute and prismatic joints combined in an RRPR sequence. The Da Vinci Endoscopic Camera Manipulator requires that both master manipulators are used simultaneously to move the camera, resembling the action of “dragging” the scene. As for the surgical instrument, only the subset of RCam joints is provided in the Unity simulation, and not the entire robot. The RCam rotates around its RCM axes (Figure 6A) based on the delta displacements of the two haptic devices moving in the same direction (Figure 6B). This allows the user to control 3 DoF out of 4, i.e., move the camera right/left, up/down, and zoom in/out. The last DoF is the camera roll rotation, which is controlled by moving the two controllers in opposite directions along the same axis. For example, as shown in Figure 6B in the bottom right image, one controller moves up while the other moves down.

Thus, the camera rotation control is achieved by: (i) comparing the delta displacements to verify that they are parallel and discordant, (ii) exploit the delta displacement as rotational magnitude and (iii) computing cross-products to find the direction of rotation. This is described by the formulas:

(3) $$\Delta p_{R}=p_{1_{R}}-p_{0_{R}}$$

(4) $$\begin{array}{rl} & c_{R}=p_{1_{R}}\times p_{0_{R}};\;if\;\left\{ \begin{array}{cc} c_{R_{z}}>0\; & \text{counterclockwise}\;\\ c_{R_{z}}<0\; & \text{clockwise}\; \end{array}\right. \end{array}$$

where $\Delta p_{R}$ is the delta displacement, $c_{R}$ is the result of the cross-product (3×1 vector), while $p_{1_{R}}$ and $p_{0_{R}}$ are the cartesian positions (3×1 vectors) in two consecutive time instants for the right Geomagic Touch instrument. The same goes for $\Delta p_{L}$ and $c_{L}$, which are calculated with $p_{1_{L}}$ and $p_{0_{L}}$. When computing the cross product between two three-dimensional vectors, such as $p_{1_{R}}$ and $p_{0_{R}}$, the result is a vector $c_{R}$ that is perpendicular to the plane defined by $p_{1_{R}}$ and $p_{0_{R}}$. This resulting vector can be interpreted as the axis of rotation that brings $p_{1_{R}}$ to $p_{0_{R}}$. The z-component of the resulting vector ($c_{R_{z}}$) provides crucial information about the direction of rotation. In particular, if $c_{R_{z}}$ and $c_{L_{z}}$ are positive, then the rotation is counterclockwise; otherwise, it is clockwise. Inverse kinematics surgical instruments and camera control allow a scaling factor to control the motion speed in the simulation. This factor can be set by an external tablet communicating with the node via MQTT (28).

### 2.3 Simulator

The training scene is implemented using Unity Engine (25), one of the most famous cross-platform game development software. Given that Unity does not inherently support the simulation of robots, unlike Coppelia (V-REP), we exploited Unity Empty GameObjects to build the virtual kinematic chain following the Da Vinci DH tables (Table 1).

Compared to (21), which simulates and visualizes the entire Da Vinci robot, our study simulates the whole robot but only visualizes the last segment of the surgical instrument (Figure 5B). Specifically, we group the first four joints (3 revolute and 1 prismatic) into {RB}, as illustrated in Figure 5B. These joints are responsible for the RCM condition, with the RCM positioned at the origin of {RB}. We did this to simplify the simulation by reducing the number of components to be rendered.

To control the whole virtual robot kinematic chain, we retrieve the six joint values (J) solving the inverse kinematic equations, computed within the Teleoperation node (Section 2.2.3). These values are used in the Unity simulation as inputs for the respective joint GameObjects.

**Figure 7:**
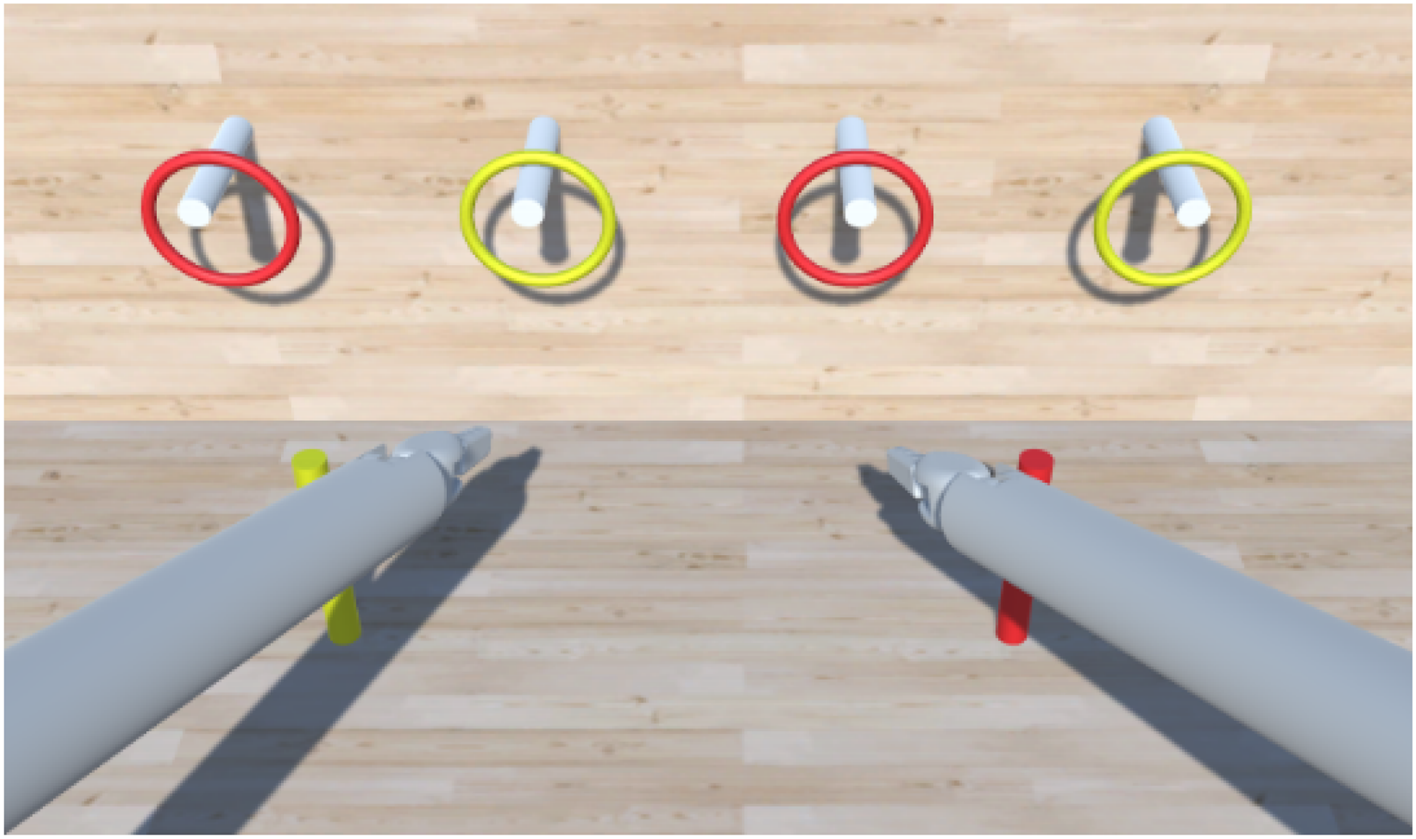
Unity scene showing the Peg Board exercise. The task is completed when all the rings are in the relative peg on the floor.

In order to test and assess the system’s usability, we implemented a Peg Board scene. As in other existing simulators, such as (29–31), the Peg Board represents one of the basic exercises for surgical training for object manipulation. As shown in Figure 7, the Peg Board exercise consists of picking up the rings on the pegs and placing them on the ground in the pegs of the corresponding colour. The scene is implemented using GameObjects and Colliders, essential to recognize a collision between two or more GameObjects. It is important to note that Unity lacks a built-in collider shape that resembles a hollow ring. Thus, it was necessary to implement multiple CapsuleColliders for each ring element, with a careful arrangement to achieve the desired shape and collision behaviour.

The Unity simulation needs the data computed by the Teleoperation node to control the joints’ movements, and these data are sent via ROS2 messages. The Ros2ForUnity plugin (32) is used to make Unity interact with ROS2 publishers and subscribers.

One of the major advantages of adopting Unity is its capacity to facilitate the creation of complex scenes, including various components such as robots, tools, objects, devices, physics and organs. This flexibility enables the rapid expansion of the simulator’s capabilities, making it a valuable choice for diverse and advanced simulations.

#### 2.3.1 Haptic feedback

The effectiveness of haptic feedback in robotic surgery is a highly debated topic (23, 24, 33, 34). Several commercial surgical robotic platforms, such as the Da Vinci robotic system, do not include haptic feedback, making the surgeon rely only on his eyesight to perform the surgery. Since this work aims to lay the foundations for creating a training platform for young surgeons, haptic feedback could be an added value in the learning phase (35, 36). As a result, an initial implementation of haptic feedback was incorporated, but its development has been limited to pliers interactions with scene elements. In detail:

1. Vibration when a ring is gripped;
2. Preventing the user from crossing surfaces with surgical instruments. These surfaces are the simulation floor, the walls and pegs;
3. Interaction feedback between the gripped ring and the pegs.

The feedback (1) is implemented by sending a force feedback of 1N when both the grippers of an instrument touch a ring. This feedback is constant and is applied for 3 ms in the positive direction of the {H} *x*-axis (Figure 5A).

Conversely, feedback (2) and (3) follow a more detailed model. This work proposes a Mass-Spring-Damper model (5).

(5) $$\Sigma F=-k_{s}x-c\dot{x}$$

All the vectors are defined in {Unity}. $k_{s}$ is the stiffness coefficient, c is the viscosity coefficient, x is the displacement from the equilibrium position (i.e., how much the EE penetrates the surface of the object), and $\dot{x}$ is the velocity of the EE. x and $\dot{x}$ (both 3×1 vectors) are provided by Unity, while $k_{s}=0.6$ and c=0.01 were found in an empirical way to ensure the sensation closely resembled contact with a rigid surface. All the feedback are sent via ROS2 messages and are received by the Haptic node, which renders the forces. The transformation between {Unity} and {H} is necessary to refer the feedback values in {H}. Some feedback has not been implemented, such as when the two surgical instruments collide or when the body of an instrument makes contact with a scene element. In Unity, the collisions are handled through Colliders elements.

## 3 Experimental validation

### 3.1 Experimental setup

Figure 1 illustrates the experimental setup. We opted for the Pro-Cart (ITD GmbH, Germany)(5,500 USD) as the commercial cart, given its availability in the lab. A lower cost cart can easily be found (e.g., the Medical Trolley OC-1T by Likaymo, 400 USD). The selected haptic devices are the Geomagic Touch (3DSystems, USA)(5,000 USD), with each stylus replaced by the Twee stylus (BBZ Srl, Italy)(1,600 USD) gripper interface, offering an additional degree of freedom to control the surgical instrument’s forceps. Although the system can display interleaved images for 3D viewing with polarized glasses or standard 2D images, we opted for the Meta Quest 2 VR headset (Meta, USA)(350 USD) to replicate a visual mode closely resembling the surgical one. Windows operating system (PC cost: 1,000 USD) was chosen based on its compatibility with the VR visor. The cost for each element of the Surgical Robot Simulator interface is indicated in brackets. Thus, the estimated total cost of the architecture components is approximately 8,400 USD if a lower cost cart is selected.

All the nodes are implemented in C++, using the ROS2 framework (37), which manages the communication of the packages. The libraries used are (i) orocos_kdl (ROS2 package) to compute the joints value through the inverse kinematic and (ii) the OpenHaptics library (3DSystems, USA) to retrieve the data from the Geomagic Touch.

### 3.2 Teleoperation accuracy assessment

The teleoperation accuracy was assessed with a displacement analysis by comparing input and output displacements between the haptic devices and the simulated instruments. The main objective is to estimate the error introduced by our teleoperation architecture (Figure 3), which comprehends input extraction, data elaboration, inverse kinematics equations and applying the results to tools simulated in Unity. Therefore, the idea is to compare the input displacement of the HIP, controlled by the Geomagic Touch, with the output displacement of the simulated EE. Both linear and angular variations have been checked by moving the Geomagic Touch HIP along its axes and measuring a set of 12 initial and final pose in both the reference systems, {H} and {RB}. The homogeneous matrix describing the pose error can be defined in compact notation as:

(6) $${err}_{P}=\left[\begin{array}{cc} {err}_{\mathrm{ang}} & {err}_{\mathrm{lin}}\\ 0 & 1 \end{array}\right]$$

where ${err}_{\mathrm{lin}}$ is a 3×1 vector representing the linear error, while ${err}_{\mathrm{ang}}$ is a 3×3 matrix indicating the angular error. ${err}_{P}$ can be further explained in:

(7) $${err}_{P}=\left({\;}^{\left\{RB\right\}}P_{1}{*}^{\left\{RB\right\}}P_{0}^{-1}\right)*{\left({\;}^{\left\{RB\right\}}T_{\left\{H\right\}}*\left({\;}^{\left\{H\right\}}W_{1}{*}^{\left\{H\right\}}W_{0}^{-1}\right)\right)}^{-1}$$

where ${\;}^{\left\{RB\right\}}P_{1}{*}^{\left\{RB\right\}}P_{0}^{-1}$ is the current pose variation in {RB}, and ${\;}^{\left\{H\right\}}W_{1}{*}^{\left\{H\right\}}W_{0}^{-1}$ is the desired pose variation in {H}.

### 3.3 Usability study

A usability study was conducted in order to evaluate the accuracy, intuitiveness and usability of the surgical robot simulator interface. This study was approved by the Regional Ethics Committee of Liguria under the protocol IIT_ADVR_TELE01, number 229/2019 - ID 4621.

#### 3.3.1 Subjects

We enrolled a total of 42 subjects. Initially, we recruited 13 expert surgeons in Robotic Surgery (RS Surg: age mean ± std: 41.7 ± 10 years, age range 32–69 years, 1 woman) and 23 controls (CTRL: age mean ± std: 49.3 ± 11.2 years, age range 32–69 years, 10 women). Then, we enrolled a new set of 6 surgeons without experience in robotic surgery (NO RS Surg: age mean ± std: 55 ± 8.7 years, age range 32–69 years, 1 woman). Inclusion criteria were: (i) not having any medical experience (i.e., no healthcare-related studies) for CTRL, (ii) being specialized surgeons with experience in robotic surgery for the RS Surg, and (iii) being specialized surgeons with no experience in robotic surgery for the NO RS Surg. All subjects completed the subsequent experimental protocol except for one control. This subject wore glasses with progressive lenses, which prevented the VR headset image from being correctly focused. Consequently, the subject was unable to perform the tasks, leading to the suspension of the experiment.

#### 3.3.2 Experimental protocol

**Figure 8:**
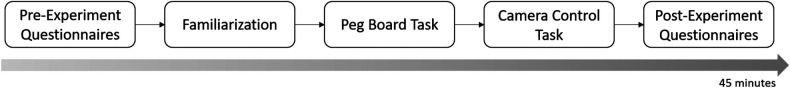
Experimental Pipeline. Each experiment consists of four macro-phases: two pre-experiment questionnaires, a familiarization phase, the execution of the two main tasks and the completion of further questionnaires after them. The average execution time of the complete pipeline is approximately 45 min.

The experimental protocol was divided into five distinct phases (Figure 8):

• Pre-experiment questionnaires;
• Familiarization;
• Peg board task;
• Camera movement task;
• Post-experiment questionnaires;

The overall duration was about 45 min.

##### 3.3.2.1 Pre-experiment questionnaires and familiarization

The pre-experiment questionnaires consist of two surveys: a demographic one and a Simulation Sickness Questionnaire (SSQ) (38). The demographic questionnaire was proposed to obtain general information about the subject (gender and age) and their frequency of use of technological devices such as smartphones and video game consoles. Finally, there were questions reserved for surgeons to determine their confidence level with laparoscopy and robotic surgery. The SSQ questionnaire is a standard validated questionnaire aimed at measuring the discomfort level resulting from exposure to a VR environment. It outlines 16 symptoms typically related to ’Simulation Sickness’ and requests the user to assess their severity on a scale from 0 to 3. This questionnaire was administered to the participants before and after the experimental phase, ensuring that any discomfort reported could be attributed to the simulation. After the pre-experiment questionnaires phase, the subjects started the familiarization phase: every user had a little time to get used to the system (5 to 10 min). At the end of the familiarization, the subject started to perform the two following tasks.

##### 3.3.2.2 Peg board task

The Peg Board task was conducted twice for each participant: once with haptic feedback enabled and once without it. The testing order was randomized for every user to mitigate potential bias introduced by using a specific system first. As shown in Figure 7, the Peg Board task consists of picking the four rings from the cylinders on the wall and placing them in the two corresponding cylinders on the floor. This experiment aimed to determine whether users controlling the system can reliably pick objects up from one location and place them at another using the gripper end effector. Moreover, comparing the presence/absence of haptic feedback aims to investigate its utility and any differences in the performance between the two cases.

##### 3.3.2.3 Camera control task

Regarding the camera movement task (Figure 6B, the user was asked to move the camera, using all 4 DoF, to align a horizontal grey line fixed on the camera plane with a green rectangle placed on the front wall in the simulation. The goal was to assess the accuracy and intuitiveness of the camera movements. The task was performed without haptic feedback as it is purely visual and does not require direct contact with any scene element.

##### 3.3.2.4 Post-experiment questionnaires

The post-experiment questionnaires consist of four surveys:

• SSQ: The first to be proposed is the SSQ to evaluate the subject’s symptoms immediately after using the simulator.
• User Experience Questionnaire (UEQ) (39): The subject is required to complete the UEQ (40), which provides a comprehensive assessment of user experience, encompassing both traditional usability metrics such as effectiveness, controllability, and learnability, as well as non-goal-oriented or hedonic factors like stimulation, fun, novelty, emotions, and aesthetics. This questionnaire comprises 26 items grouped into 6 scales: attractiveness, efficiency, perspicuity, dependability, originality, and stimulation. These scales are interrelated, with the attractiveness scale capturing the user’s overall impression influenced by values on the other 5 scales (41).
• SIM-TLX (42): The SIM-TLX questionnaire is a customized version of the NASA-TLX (43). SIM-TLX is designed explicitly for VR simulations, and it evaluates the workload associated with different tasks across various dimensions, such as mental, physical, and temporal demands, as well as complexity, distraction, and frustration levels.
• Face Validity Questionnaire: Face Validity is a custom questionnaire to assess the superficial appearance or “face value” of an instrument, such as a test, a device, or an assessment tool. Face validity allows us to gather subjective opinions on whether our simulator is helpful at first glance. The questionnaire contains multiple items, each employing a Likert scale response format with five points ranging from “very bad” to “very good.” First, there are four general items concerning the level of realism of the simulated instruments, the quality of the images, the comfort of the hardware interface, and whether haptic feedback helps or hinders. Then, four extra items were reserved for surgeons, which regard: (i) the ability to triangulate an object in the simulation, (ii) the realism of hand movements compared to robotic surgery, (iii) the comfort level of the interface compared to commercial ones, (iv) the usefulness of simulation in the acquisition of hand-eye coordination.

#### 3.3.3 Analysis

The performance data recorded during the Peg Board Task include:

• Time required to perform the task (Peg Board Execution Time, $t_{\mathrm{PB}}$)
• Number of rings transferred correctly ($n_{\mathrm{RTC}}$)
• Number of rings dropped ($n_{\mathrm{RD}}$)
• Number of instrument collisions ($n_{\mathrm{IC}}$)

We established the number of errors ($n_{\mathrm{Err}}$) in the PegBoard task as the sum of $n_{\mathrm{RD}}$ and $n_{\mathrm{IC}}$.

Regarding the Camera Control Task we recorded:

• Time required to perform the task (camera execution time, $t_{C}$)
• Alignment Error (AlignErr)

Referring to Figure 6B, we estimated the AlignErr by comparing the angle between the green rectangle and the grey line, which is the horizontal baseline. Thus, the AlignErr corresponds to the slope of the green rectangle. A binary mask is computed by segmenting the green colour on the view of the virtual camera in the simulation. Therefore, the coordinates of 2 points belonging to the rectangle mask’s upper side are automatically identified to calculate the gradient of the straight line passing between them. The AlignErr is expressed in degrees since the angular coefficient is converted into the relative slope angle with respect to the abscissa axis through the arctan operation.

Questionnaire data and performance data were compared among the groups using the non-parametric Wilcoxon-Mann-Whitney test due to the non-normal distribution of the data. Results were considered significant with a *p*-value < 0.05. All analyses were conducted using Matlab 2022b.

## 4 Results and discussion

### 4.1 Teleoperation accuracy results

The accuracy of the software chain is described in Table 2. The experiments show that the ${err}_{\mathrm{lin}}$ are in the order of magnitude of $10^{-2}$ mm, which is reasonable considering the simulation context and the fact that the resolution of the Touch’s position sensors is 0.055 mm. The ${err}_{\mathrm{ang}}$ is computed as a rotation matrix and converted to the ZYX Euler angles convention to simplify the interpretation of the actual angle error. The order of magnitude is $10^{-2}$ degrees.

**Table 2:** Teleoperation accuracy results.

	Mean ± std	RMSE
${err}_{\mathrm{lin}}$	(0.007,0.009,0.010)±(0.032,0.030,0.043)	0.045
${err}_{\mathrm{ang}}$	(0,−0.008,0.005)±(0.011,0.024,0.015)	0.020

### 4.2 Peg board task

All subjects managed to complete the task by successfully inserting of all four rings, achieving a $n_{\mathrm{RTC}}$ of 4.

**Figure 9:**
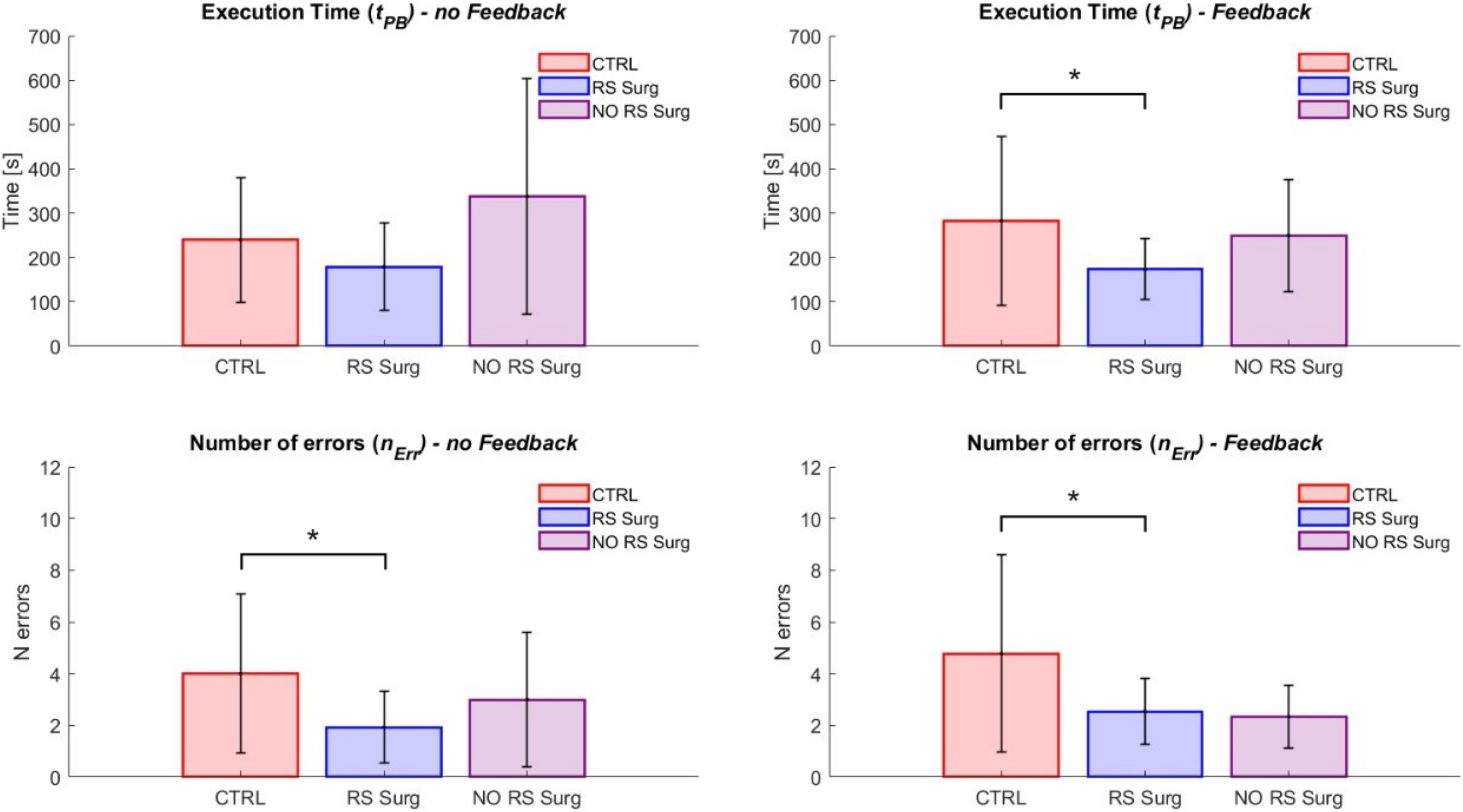
Peg board task results. The graphs illustrate the mean and standard deviation of the execution times ($t_{\mathrm{PB}}$) and the number of errors made ($n_{\mathrm{Err}}$). The two graphs on the left show the outcomes in the scenario without feedback, while the ones on the right display those with haptic feedback. (*) means a statistical difference was found with a p<0.05.

Figure 9 shows the results related to the execution times and number of errors in the Peg Board task. We examined potential differences between experts (RS Surg) and controls (CTRL) in the condition without haptic feedback. Regarding the time required to complete the task (mean ± std; CTRL = 239.6±140.1 s; RS Surg = 179.0±98.5 s), no significant difference was observed (Figure 9, left). However, in terms of number of errors (mean ± std; CTRL = 4.0±3.1; RS Surg = 1.9±1.4), a significant difference was evident with a p=0.0446. In surgery, the execution time is crucial as it impacts the procedure’s total duration and, therefore, its costs (44). Nonetheless, the number of errors made carries even more weight as it directly impacts patient health and can lead to complications and procedural delays. In this regard, the results are promising, demonstrating RS Surg’s higher proficiency and attentiveness. Robotic surgeons already possess hands-on experience with the robot, giving them an edge when using a simulator designed to emulate it. This enables them to accomplish the task with significantly fewer errors than the control group and with shorter average completion times. The superior performances of the RS Surg represent a good indicator of the fidelity of our simulator.

Given the significant difference between CTRL and RS Surg, we decided to investigate whether we could distinguish between the performances of robotic and non-robotic surgeons. Therefore, we compared RS Surg with NO RS Surg and found no significant differences in terms of either time (mean ± std; NO RS Surg = 337.8±264.9 s; RS Surg = 179.0±98.5 s) or number of errors (mean ± std; NO RS Surg = 3.0±2.6; RS Surg = 1.9±1.4). In this case, RS Surg exhibits superior performance on average compared to NO RS Surg, although not to a degree where significant differences are found. One potential factor could be the simplicity of the task at hand. Hence, evaluating both groups’ performances on a more demanding task would be interesting.

In the haptic feedback condition, we assessed the performance of both CTRL and RS Surg (Figure 9, right). We found a significant difference in both time (mean ± std; CTRL = 282.5±190.7 s; RS Surg = 173.3±68.3 s; p=0.0328) and the number of errors (mean ± std; CTRL = 4.8±3.8; RS Surg = 2.5±1.3; p=0.0202). Instead, no significant differences were revealed for these two parameters when comparing robotic surgeons to non-robotic surgeons (time: mean ± std; NO RS Surg = 249.7±125.7 s; RS Surg = 173.3±68.3 s; errors: NO RS Surg = 2.3±1.2; RS Surg = 2.5±1.3). These results confirm those previously observed in the scenario without feedback. Finally, we did not find significant differences within any group when comparing performances between tasks with and without feedback. This suggests that feedback might have a minimal impact on executing fundamental tasks, and its significance should be reconsidered for more complex tasks.

### 4.3 Camera control task

**Figure 10:**
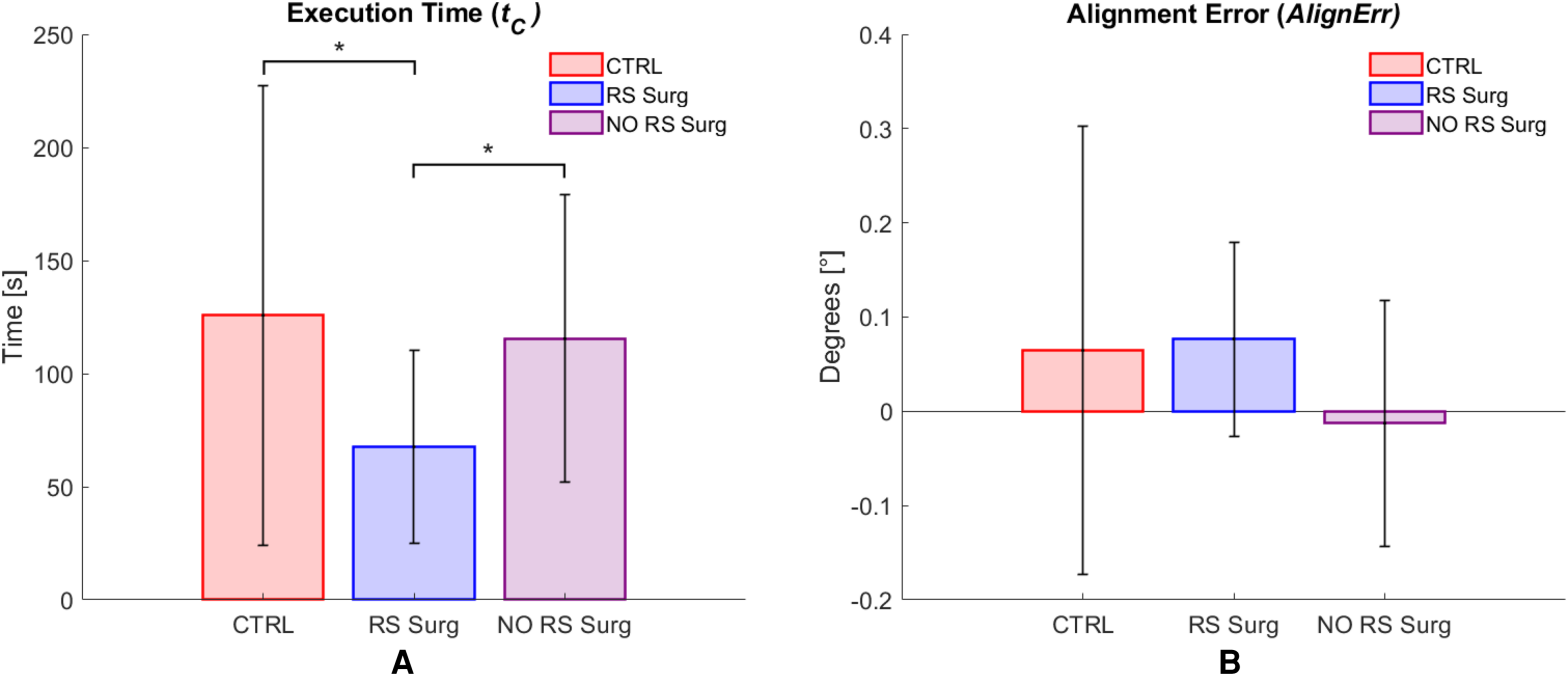
Camera control task results. Average values per group and standard deviation are reported. (**A**) Execution time ($t_{C}$) results; (**B**) Alignment error (AlignErr) results. (*) means a statistical difference was found with a p<0.05.

Figure 10 presents the results related to the camera task. Regarding the hypothesis of discerning between robotic surgeons and controls, the outcomes indicate that a significant difference was found in terms of time (CTRL vs. RS Surg: mean ± std; CTRL = 125.7±101.7 s; RS Surg = 67.7±42.8 s; p=0.0185), but no significant difference was observed in terms of AlignErr (CTRL vs. RS Surg: mean ± std; CTRL = 0.06±0.24; RS Surg = 0.08±0.10). We also investigated differences between robotic surgeons and non-robotic surgeons, and found significant differences in terms of time (NO RS Surg vs. RS Surg: mean ± std; NO RS Surg = 115.5±63.5 s; RS Surg = 67.7±42.8 s; p=0.0462), but no significant difference was observed in terms of AlignErr (NO RS Surg vs. RS Surg: mean ± std; NO RS Surg = −0.01±0.13; RS Surg = 0.08±0.10). We expected to find no statistical differences on AlignErr in both tests since the task (Figure 6B) was to achieve the best alignment without any time constraints. Therefore, some subjects obtained excellent results despite investing more time. Hence, we evaluate execution time as the most crucial variable for this task. As evidenced by the tests, the RS Surg group outperformed the other groups in terms of speed, probably thanks to their prior knowledge.

### 4.4 Questionnaires

The analysis of the SSQ data did not reveal any discomfort resulting from the use of the simulator; in fact, none of the participants indicated any worsening on the 16 symptoms tracked.

**Figure 11:**
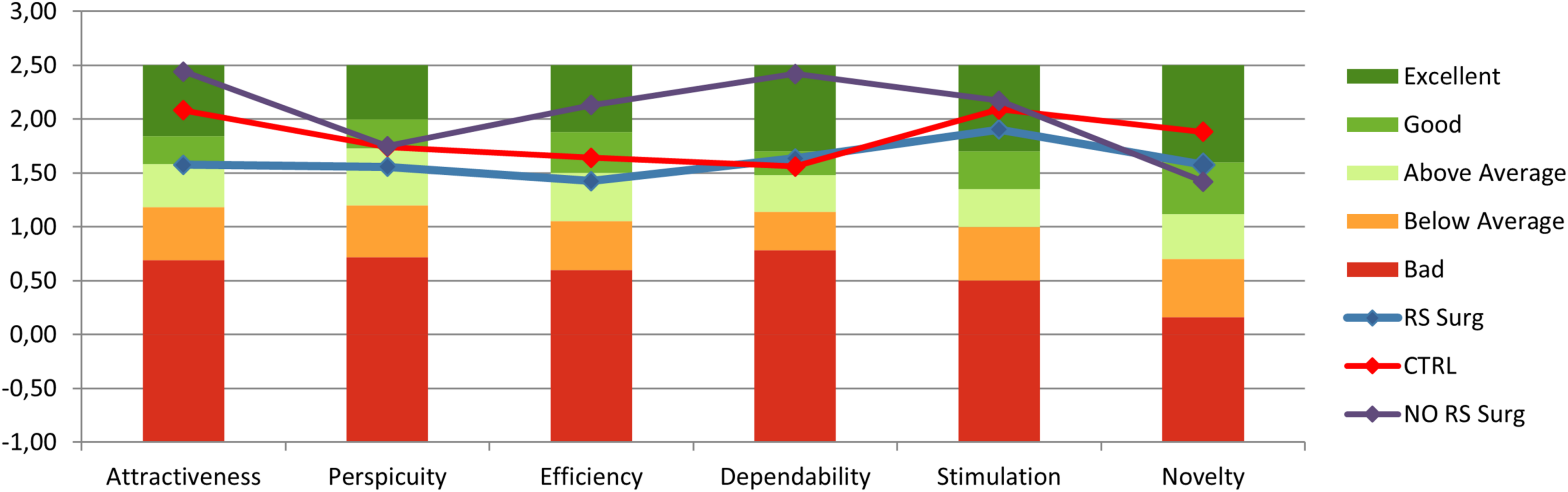
User experience questionnaire scales results (Mean) for each group, range between −1 and +3.

The outcomes of the user experience questionnaire (Figure 11) reveal values exceeding 1.5 (ranging from −1 to +3), indicating a favorable user experience during simulator usage. Initially, no notable disparities were detected between CTRL and RS Surg (Table 3), with both groups reporting a positive user experience. Similarly, upon comparing RS Surg with NO RS Surg (Table 3), no significant differences emerged, underscoring consistent positive user experience findings across both comparisons. Overall, RS Surg proved to be the most critical population, giving the lowest ratings on average, likely influenced by certain limitations of the simulator discussed in the following paragraphs. Despite this, the scores almost always fall into the “Good” range.

**Table 3:** User experience questionnaire scales results (mean ± std), range between −3 and +3.

	CTRL	RS Surg	NO RS Surg
Attractiveness	2.083±0.775	1.577±0.954	2.444±0.608
Perspicuity	1.739±1.015	1.558±0.742	1.750±1.109
Efficiency	1.636±0.883	1.423±0.872	2.125±0.830
Dependability	1.557±0.842	1.635±0.707	2.417±0.539
Stimulation	2.091±0.849	1.904±1.000	2.167±0.830
Novelty	1.875±0.933	1.577±1.072	1.417±1.233

**Figure 12:**
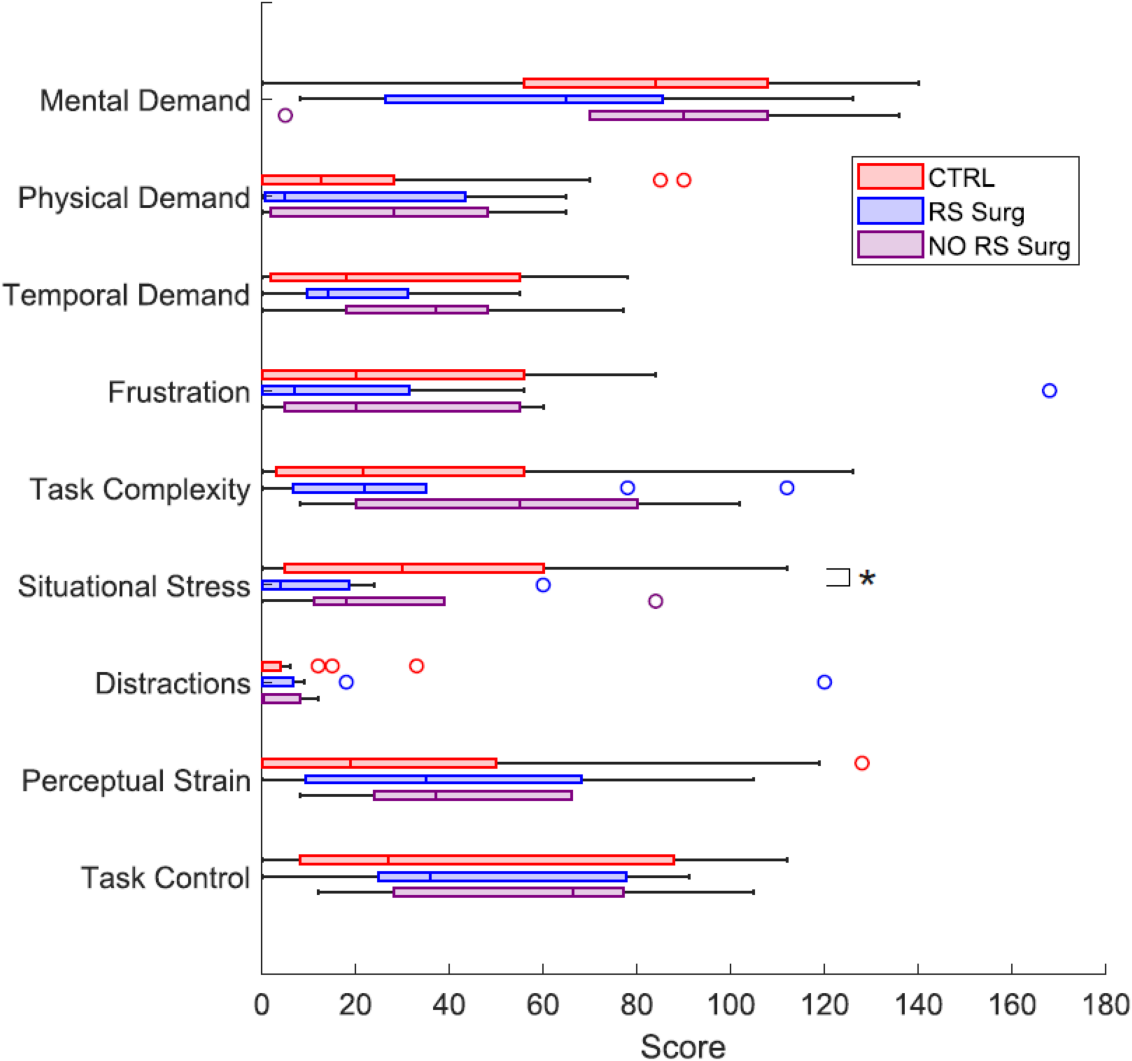
Box plots representing the workloads of the 9 dimensions of the SIM-TLX questionnaire, compared between CTRL, RS Surg and NO RS Surg. (*) means a statistical difference was found with a p<0.05.

Regarding the SIM-TLX, we calculated the workloads associated with each dimension and generated the corresponding box plots, as shown in Figure 12. Initially, we evaluated potential differences in dimensions between CTRL and RS Surg by employing the Wilcoxon rank sum test on the median, as the scores are discrete data. The test shows a significant difference in the “Situational Stress” dimension (median,[Q1,Q3]; CTRL = 30,[5,60]; RS Surg = 4,[0,18.5], p=0.021). The difference is reasonable, considering this is a primary task for the RS Surg, who are accustomed to significantly more demanding circumstances during their work. We would also have expected a difference between the two populations on the Mental Demand dimension; despite this, we obtained p=0.15. A significant difference might be observed with a more complex task. The other dimensions did not reveal significant differences, although the average workloads of the RS Surg tend to be lower than those of the CTRL, except for the “Perceptual Strain.” Subsequently, similar to the comparison made for the Peg Board and Camera task, we proceeded to explore the diverse effects of the workload associated with different levels of experience, so we conducted the Wilcoxon test between RS Surg and NO RS Surg. However, we found no statistical difference in any dimension in this case. This leads us to think that the simulator is equally accessible to every subject, whether with or without previous experience.

A qualitative analysis is presented regarding the Face Validity Questionnaire. We observed the subjects’ responses while maintaining the division into the three groups and extracted the median score. We found that for the items “realism of the simulated tools” and “comfort of the hardware interface,” all three groups have a median score of 4 (“good”) out of 5. Regarding “image quality,” RS Surg have a value of 3, while CTRL and NO RS Surg have 4. In the items specific to RS Surg participants, a notable rating of 2 (“bad”) out of 5 is recorded under the category of “realism of hand movements compared to robotic surgery.” In this case, surgeons complained about the Geomagic Touch’s movement range, which is much narrower than Da Vinci’s, which they are used to. Indeed, the horizontal range of motion for the Geomagic Touch is approximately half of the Da Vinci one. In our setup, the clutch pedal is employed more often to compensate for this difference in range. However, based on feedback from surgeons, this pedal is less utilized with the Da Vinci system. Moving to the “ability to triangulate an object in the simulation,” they provided a median value of 3, which reflects the influence of the previously mentioned issue. Concerning the “comfort level of the interface compared to commercial ones,” their response yielded a median score of 3. Some participants noted issues with the tweezers, which were perceived as more slippery and challenging to adjust than Da Vinci’s. Others raised concerns about the absence of gravity compensation on the controllers. Probably, these factors also influenced the evaluation in the UEQ, as the scores provided by the RS Surg were slightly lower than those of the other two groups. However, despite these challenges, they rated the “usefulness of simulation in the acquisition of hand-eye coordination” with a score of 4, which may indicate the simulator presents high efficacy for training purposes. Finally, regarding haptic feedback, all three groups found it beneficial, indicating a median score of 4. This result may seem controversial, as we found no statistical differences in performance with or without feedback. Despite this, the impression is that users appreciate the presence of additional help during the tasks. It would be valuable to conduct further investigations with more challenging exercises and to increase the number of subjects.

## 5 Conclusions

This paper presents an affordable user interface integrated with a surgical robot simulator. As detailed in Section 3.1, the total cost of the architecture is approximately 8,400 USD, making it comparable to other affordable solutions. Regarding the evaluation of effectiveness, we relied on the results of the usability study.

Our system is designed to be generic and has the potential to simulate the user interfaces of a wide range of surgical robots available in the market. The simulator can be used with either a VR headset or a monitor. In the monitor mode, users can select between 2D images or interleaved images for 3D viewing with polarized glasses. In this study, we decided to simulate the Da Vinci robot, which is the most used in robotic surgery, exploiting Unity. The flexibility of this platform allows the development of increasingly complex and realistic scenes, encouraging further developments of the simulator. Moreover, leveraging the modularity of ROS2 nodes, we have previously used the interface to command a real robotic arm (26) simply by adjusting the robot kinematics within the Teleoperation node.

The system accuracy assessment demonstrates that the proposed interface complies with the positioning error requirements (<1 mm) for a surgical robot platform, with errors in the order of magnitude of $10^{-2}$ mm. The usability study, which involved recruiting three groups: controls, robotic and non-robotic surgeons, demonstrates the validity of the simulator. As demonstrated in Sections 4.2, 4.3, the robotic surgeons exhibited significantly superior performances compared to the control group, indicating the transferability of their expertise to our simulator. Regarding the comparison between robotic and non-robotic surgeons, we did not find significant differences, although the RS Surg generally performed better on average. Since robotic surgical skills are highly specialized, further developments are needed to design more challenging tasks that can better showcase these skills. Thus, incorporating new metrics will enable us to evaluate different levels of surgeon experience during training. Additionally, this will provide an opportunity to reassess the haptic feedback, as the current experiments did not reveal significant performance differences. On the other hand, proposing two basic tasks allowed us to demonstrate the usability and intuitive control of the simulator; even users with minimal experience could accomplish both tasks after becoming familiar with the system. In support of this thesis, the positive results of the SSQ and UEQ (Section 4.4) demonstrate users’ satisfaction with the simulator.

It is important to acknowledge that the simulator has drawbacks. Robotic surgeons have pointed out that the range of motion of the Geomagic Touch is limited compared to that of the Da Vinci, and the tweezers have different handles. Consequently, the experience is less realistic than using the actual robot in the operating room. However, observing the face validity results (Section 4.4), they are willing to accept these compromises associated with adopting low-cost technologies. They believe this simulator can train novice surgeons in practising fundamental robotic surgery manoeuvres, such as wrist movements, coordinating with the clutch pedal, and controlling the camera. In conclusion, the surgeons endorsed our idea: the development of an affordable simulator, which opens up the possibility of establishing training programs in robotic surgery within medical schools and universities, is needed to make such training accessible to a broader audience.

## Data Availability

The raw data supporting the conclusions of this article will be made available by the authors, without undue reservation.
